# Trajectories of distress from pregnancy to 15-months post-partum during the COVID-19 pandemic

**DOI:** 10.3389/fpsyg.2023.1104386

**Published:** 2023-03-31

**Authors:** Jennifer E. Khoury, Marc Jambon, Lauren Giles, Leslie Atkinson, Andrea Gonzalez

**Affiliations:** ^1^Department of Psychology, Mount Saint Vincent University, Halifax, NS, Canada; ^2^Department of Psychology, Wilfrid Laurier University, Waterloo, ON, Canada; ^3^Department of Psychology, Metropolitan Toronto University, Toronto ON, Canada; ^4^Department of Psychiatry and Behavioural Neurosciences, McMaster University, Hamilton, ON, Canada; ^5^Offord Centre for Child Studies, McMaster University, Hamilton, ON, Canada

**Keywords:** COVID-19, postpartum depression, pregnancy, parent mental health, perinatal stress

## Abstract

**Background:**

The COVID-19 pandemic has particularly burdened pregnant and postpartum women. It remains unclear how distress levels of pregnant and postpartum people have changed (or persisted) as the pandemic continues on and which factors may contribute to these trajectories of distress.

**Methods:**

This longitudinal study included 304 pregnant people, who were followed during pregnancy, 6-weeks, 6-months and 15-months postpartum. At each time point, a latent “distress” factor was estimated using self-reported depressive symptoms, anxiety symptoms, and stress. Reported negative impact of COVID-19 and social support were assessed during pregnancy as risk and protective factors related to distress. Second-order latent growth curve modeling with a piecewise growth function was used to estimate initial levels and changes in distress over time.

**Results:**

Mean distress was relatively stable from the pregnancy to 6-weeks postpartum and then declined from 6-weeks to 15-months postpartum. Higher education, greater social support, and lower negative impact of COVID-19 were associated with a lower distress during pregnancy. Unexpectedly, negative impact of COVID-19 was associated with a faster decrease in distress and more social support was associated with a greater increase in distress from pregnancy to 6-weeks postpartum. However, these effects became non-significant after controlling for distress during pregnancy.

**Conclusion:**

Findings indicate high but declining levels of distress from pregnancy to the postpartum period. Changes in distress are related to social support and the negative impact of the pandemic in pregnancy. Findings highlight the continued impact of COVID-19 on perinatal mental health and the need for support to limit the burden of this pandemic on pregnant people and families.

## Introduction

The past 2 years of the COVID-19 pandemic have taken a toll on the lives of many. The combination of isolation from family and friends, economic and educational turmoil, and bereavement, have contributed to exorbitant elevations in mental illness around the globe (Salari et al., 2020; Bueno-Notivol et al., 2021). It is essential to understand the continued psychological impact of the pandemic over time, to reduce enduring psychological effects in the years to come. It is particularly important to assess the impact on psychologically vulnerable populations. Pregnancy and the early postpartum period are naturally marked by increased vulnerability to stress and mental health problems (Woody et al., 2017), which has been exacerbated during the COVID-19 pandemic (Yan et al., 2020; Fan et al., 2021; Sun et al., 2021). However, it remains unclear how mental health problems of pregnant and postpartum people change (or persist) as the pandemic continues.

Prenatal and postpartum elevations in mental health problems and distress can have direct and indirect consequences to the mother’s health, the child’s development, as well as the parent–child relationship (Beck, 1998; Kingston et al., 2012; Kingston and Tough, 2014; Caparros-Gonzalez et al., 2021). Much of the research on perinatal maternal distress, including this study, focuses on self-reported psychological distress, including reported depressive symptoms, anxiety symptoms, and stress, rather than clinically diagnosed disorders (also see Khoury et al., 2022). Meta-analytic evidence indicates that pregnant and postpartum people experienced high rates of distress (70%), anxiety symptoms (34–42%) and depressive symptoms (25–31%) in the initial pandemic period (Yan et al., 2020; Fan et al., 2021; Sun et al., 2021). Pregnant people have experienced more severe depressive symptoms during the COVID-19 pandemic compared to non-pregnant people (López-Morales et al., 2021). Furthermore, people who were pregnant during the pandemic experienced higher levels of depressive symptoms compared to a matched sample of individuals who were pregnant prior to the pandemic (King et al., 2021).

Several factors increase risk for developing elevated prenatal and postpartum depressive, anxiety, and stress symptoms (Hutchens and Kearney, 2020). Elevated distress symptoms in pregnant people during the pandemic are associated with low levels of social support (Lebel et al., 2020; Khoury et al., 2021b; Filippetti et al., 2022), financial difficulties (Lebel et al., 2020; Sherin et al., 2021), less adaptive coping strategies (Khoury et al., 2021a), reduced exercise (Gildner et al., 2020), and more negative cognitive appraisal of the impact of the pandemic (Khoury et al., 2021b). In addition, a recent review indicated that low social support during pregnancy, as well as higher levels of prenatal stress, depressive symptoms and anxiety symptoms increased the likelihood of developing maternal postpartum depression during the pandemic (Doyle and Klein, 2020). However, it remains unclear which factors are associated with elevations in perinatal distress across several months of the pandemic.

Furthermore, emerging data indicates long-term adverse mental health and distress effects on new mothers during the pandemic. A handful of longitudinal studies have shown that people who were pregnant before the pandemic experienced increased rates of stress, depressive symptoms, and anxiety symptoms in the postpartum pandemic period (Naurin et al., 2020; Wang et al., 2020; Boekhorst et al., 2021; Hamidia et al., 2021). In addition, other studies have followed participants from pregnancy to the postpartum, during the pandemic, and have shown elevated rates of mental health problems. For example, Gluska et al. (2022) showed that women in Israel experienced increased levels of depressive symptoms from 3- to 6-months postpartum during the pandemic. Additionally, a study of pregnant people in Quebec, Canada, found that prenatal maternal distress was associated with distress at 2-months postpartum, and that postpartum distress mediated the association between prenatal distress and early infant social–emotional development (Duguay et al., 2022). Furthermore, Fernandes et al. (2022) conducted a 3-wave longitudinal study, spanning from the third trimester of pregnancy until 6-months postpartum. They found that lockdown measures, lower social support, and higher levels of depressive symptoms during pregnancy predicted trajectories of depression over time. Existing longitudinal studies have not examined the impact of the pandemic on mental health problems beyond the first 6 months postpartum. The present study extends this research by following pregnant people into the second year of the postpartum period.

### The present study

Using a longitudinal approach, the present study assessed pregnant and postpartum people across four time points during the COVID-19 pandemic: pregnancy (T1), 6-weeks postpartum (T2), 6-months postpartum (T3) and 15-months postpartum (T4). The primary goal of the study is to assess pregnancy-specific risk for perinatal distress levels over the course of the pandemic. The present study had three aims: (1) to describe average (mean) levels of perinatal distress (depressive, anxiety, and stress symptoms) at four time points, spanning nearly 2 years of the COVID-19 pandemic, (2) to examine rate of change in distress symptoms across the perinatal period, and (3) to assess whether the negative impact of the COVID-19 pandemic and social support during pregnancy were associated with initial levels or change in distress across the COVID-19 pandemic. We hypothesized that levels of distress would be highest during the early phase of the pandemic, coinciding with pregnancy (T1), and would decline steadily thereafter. We also hypothesized that higher social support during pregnancy would predict lower levels of distress over time and that more negative COVID-19 experiences would predict higher levels of distress over time.

## Methods

### Participants and study design

A total of 304 pregnant people from Ontario, Canada participated in the first survey (T1) for the COVID-19 and Wellbeing During Pregnancy Study. Participants completed online questionnaires at four time points (T1: pregnancy, *n* = 304; T2: 6-weeks postpartum, *n* = 265, T3: 6-months postpartum, *n* = 180; T4: 15-months postpartum, *n* = 190). Of note, in addition to these four time points, we invited participants to complete surveys during each trimester of pregnancy (e.g., those who began the study (T1) in their first trimester were invited to complete surveys in the second and third trimester). Given that different participants completed a varied number of pregnancy surveys, depending on their gestational age at study onset (T1), we do not include data across multiple trimesters in our analyses. All pregnancy data used in the current study was taken from T1, at the onset of they study (summer 2020).

T1 surveys were completed between June and August 2020, T2 surveys were completed between July 2020 and May 2021, T3 surveys were completed between February 2021 and October 2021, and T4 surveys were completed between October 2021 and June 2022. For context, a state of emergency was declared by the provincial government of Ontario three times between March 17, 2020, and April 7, 2021, which included over 300 days of lockdown. T1 data collection occurred, on average, 111 days after the first state of emergency was declared in Ontario. After T1, 6 participants withdrew (4 due to miscarriage, 2 for undisclosed reasons), and 33 participants did not respond to follow-up survey requests. Attrition between T2 and T4 was due to participants not responding to follow-up survey requests. Initial recruitment was conducted through social media advertisements, pamphlets distributed to midwifery groups, and word of mouth. At T1, inclusion criteria were that individuals (1) live in Ontario, Canada, (2) read and write English, (3) be at least 18 years of age, and (4) be ≤36-weeks’ gestation.

### Measures

#### Distress

##### Depressive symptoms

The 10-item Center for Epidemiologic Studies Depression Scale (CES-D; Anderson et al., 1994) was used to measure depressive symptoms over the past 7 days. Responses range from 0 “*rarely or never (less than 1 day)*” to 3 ‘*most or all of the time (5–7 days)*’. The CES-D total score ranges from 0 to 30; a cut off score of 10 or higher indicates the presence of clinically significant depressive symptoms (Anderson et al., 1994). The CES-D has shown good reliability and validity in pregnant and postpartum samples (Beeghly et al., 2003). The CES-D showed good internal consistency in the current sample across T1-T4 (Cronbach’s *α* range = 0.87–0.87).

##### Generalized anxiety symptoms

The 7-item Generalized Anxiety Disorder-7 (GAD-7) scale was used to measure GAD symptoms occurring in the past 2 weeks (Spitzer et al., 2006). Responses range from 0 “*not at all*” to 3 “*nearly every day*.” The GAD-7 total score ranges from 0 to 21; scores between 0 and 4 indicate no anxiety symptoms, scores between 5 and 9 indicate mild anxiety symptoms, scores between 10 and 14 indicate moderate anxiety symptoms, and scores between 15 and 21 indicate severe anxiety symptoms. The GAD-7 has shown strong psychometric properties in pregnant and postpartum samples (Simpson et al., 2014; Zhong et al., 2015; Gong et al., 2021) and demonstrated good internal consistency in the current sample across T1-T4 (Cronbach’s *α* range = 0.88–0.90).

##### Perceived stress

The 10-item Perceived Stress scale (PSS; Cohen and Williamson, 1988) assesses perceptions of stress over the past month, with responses ranging from 1 “*never*” to 4 “*very often*.” The PSS total score ranges from 0 to 40; scores between 0 and 13 indicate low stress, scores between 14 and 26 indicate moderate stress, and scores between 27 and 40 indicate high levels of stress (Cohen and Williamson, 1988). The PSS had good internal consistency in the current sample across T1-T4 (Cronbach’s *α* range = 0.90–0.91).

#### Risk and protective factors

##### COVID-19 impact

The subjective and objective impact of the COVID-19 pandemic was assessed. Based on prior research indicating that the subjective impact (appraisal) of stressors affects pregnancy and child outcomes (Cao-Lei et al., 2015; Moss et al., 2017; Simcock et al., 2017; Khoury et al., 2021a,b), participants were asked “Taking everything about COVID-19 into account, the effects of COVID-19 on me and my household have been,” ranging from 1 (*very positive*) to 5 (*very negative*). Higher ratings indicate a more negative subjective impact of COVID-19 during pregnancy (T1). In addition, the objective impact of the COVID-19 pandemic was assessed through eleven questions, on a scale of 1 (*Not at all*) to 7 (*A lot*). Participants reported how much they have experienced social isolation (1 item), relationship difficulties (2 items), financial changes (6 items), risk of COVID-19 infection (1 item), and difficulty finding childcare (1 item) related to the pandemic. For additional detail, see Khoury et al. (2021a,b). The 11 objective COVID impact questions were averaged to create a subscale. The subjective COVID subscale was rescaled to be on a 7-point scale (to match the objective COVID scale). After rescaling, the objective and subjective impact subscales were averaged, to create an overall COVID impact summary score (higher numbers indicate more negative COVID-19 experiences).

##### Social support

To assess social support from significant others, family members, and friends during pregnancy, participants completed the 12-item Multidimensional Scale of Perceived Social Support (MSPSS; Zimet et al., 1988). Item ratings range from 1 (*very strongly disagree*) to 7 (*very strongly agree*). The MSPSS total score is an average composite of all 12 items, ranging from 1 to 7. The MSPSS has strong psychometric properties in pregnant and postpartum samples (Mirabzadeh et al., 2013; Carlsson et al., 2015). The MSPSS showed good internal consistency in the current sample during pregnancy (Cronbach’s *α* range = 0.95).

##### Sociodemographic characteristics, pregnancy factors, and additional COVID-19 experiences

Participants reported sociodemographic characteristics at T1, including maternal age, race/ethnicity, education, income, parity, and gestational age at T1. In addition, occurrence of gestational diabetes or miscarriage, at any point in pregnancy, were reported. At T1, participants reported a range of COVID-19 related experiences, including a positive COVID-19 result. Number of days between the state of emergency declaration (March 17, 2020) and T1 survey completion was also calculated.

### Statistical analyses

We conducted the analyses in three separate phases. First, descriptive statistics and preliminary correlations among study variables were calculated using SPSS 27. Bivariate correlations were examined to determine relevant covariates (based on prior research) to include in the subsequent models. Potential covariates were included in LGCM analyses only if there were significant bivariate correlations with distress outcomes. Specifically, we examined sociodemographic variables including maternal age, education level, income, race, and marital status, as well as number of children. We also assessed pregnancy and COVID-related variables as potential covariates, including gestational age at the time of T1, gestational diabetes, miscarriage, and number of days since state of emergency declaration. Continuous predictor/auxiliary variables were mean-centered prior to entry into the models. All subsequent analyses were conducted in Mplus 8.4 using full information maximum likelihood (FIML) estimation with robust standard errors (MLR) to account for missing data (Collins et al., 2001). FIML estimation utilizes all available data to derive parameter estimates and reduces bias in missing data compared to other methods of handling missing data (e.g., listwise deletion, mean substitution; Enders and Bandalos, 2001).

We next tested a measurement model using longitudinal confirmatory factor analysis (CFA). Ratings of depression, anxiety, and stress were rescaled onto the same 7-point metric (range = 1–7) and treated as observed indicators of a latent “distress” factor at each time point (four total: pregnancy, 6-weeks postpartum, 6-months postpartum, 15-months postpartum). We used the effects coding method to scale the latent factors (Little et al., 2006) and allowed item-specific residual errors to covary over time. With the effects coding method, latent factor means represent the optimally weighted average across the three indicators, and the latent variances represent the average variability in scores across the indicators. We evaluated longitudinal measurement invariance by comparing the fit an unconstrained model to models with equality constraints placed on factor loadings (metric invariance) and intercepts (scalar invariance). We considered changes in comparative fit index (CFI) values ≤0.010 and root mean squared error of approximation (RMSEA) values ≤0.015 as evidence that the measures were invariant over time (Little, 2013).

We then tested a series of latent growth curve models (LGCM) to examine changes in latent levels of distress over time. First, we used theory, descriptive statistics, and model fit comparisons to determine the optimal growth function (e.g., linear vs. piecewise). We first estimated a linear LGCM with the intercept centered at the first measurement occasion, and the loadings for the slope parameters set to 0, 1, 2, 3. With these constraints, the intercept mean represented the average level of distress during pregnancy, and the slope factor mean represented the average amount of estimated mean change in distress between each subsequent assessment. The intercept and slope variances represent individual differences in initial levels and rates of change, respectively. We then tested a piecewise LGCM with one intercept (centered at T1 during pregnancy) and two linear slopes. Based on the pattern of descriptives across the four time points, and the fact that childbirth is a salient event that can alter the trajectory of psychological processes, we placed the “knot” or “turning point” at T2 (i.e., 6-weeks post-partum). The loadings for the first slope construct were set to 0, 1, 0, 0, and the loadings for the second slope were set to 0, 0, 1, 2. Thus, the mean of slope 1 describes the average amount of change in distress from pregnancy to 6-weeks post-partum, whereas the mean of slope 2 describes the average amount of change in distress across each time point from T2 (6-weeks postpartum) to T4 (15-months postpartum). We then tested a second unconditional model that included negative impact of COVID-19 and social support as covariates to examine bivariate associations with initial distress levels (intercept) and changes in distress (slope[s]).

In a final conditional growth model, we regressed the intercept and slope(s) onto the predictors to disentangle their unique effects on initial levels and growth. Model fit was assessed using a combination of chi-square values, CFI, RMSEA, and Standardized Root Mean Square Residual (SRMR). Values >0.90 for CFI, and <0.08 for RMSEA and SRMR are considered indicative of acceptable model fit (Little, 2013). The Satorra-Bentler scaled chi-square difference test was used to compare the fit of nested models (Satorra, 2000).

### Missing data

Participants who completed T2 did not differ from those who did not on parent age, race, ethnicity, income, or number of children. However, participants who entered the study earlier in their pregnancy were less likely to complete T2 (*t*(302) = 2.54, *p* < 0.05), T3 (*t*(302) = 2.17, *p* < 0.05), and T4 (*t*(302) = 2.07, *p* < 0.05). Participants who had lower income were also less likely to complete T3 (*t*(295) = −2.91, *p* < 0.01) and T4 (*t*(295) = −3.03, *p* < 0.01). Lastly, those who were not in a romantic relationship (*t*(302) = 2.46, *p* < 0.05) were also less likely to complete T3. No other significant differences were found in participants who completed all time points and those who did not. Participants who completed all assessments did not differ from those who did not complete T2-T4 on perceived stress, anxiety symptoms, depressive symptoms, social support or COVID stress at T1 (*p*s range 0.07–0.86). The finding that longitudinal attrition was systematically associated with measured variables suggests that the data could be reasonably assumed to be missing-at-random (MAR). To support missing data estimation under the MAR assumption, weeks gestation at study entry, relationship status, and income were included as auxiliary variables (i.e., covariates that used to inform the estimation of missing data but are not statistically controlled for in the analyses) or control variables in all models (Enders, 2012; Rioux and Little, 2021). Variables that were not significantly associated with study outcomes were treated as auxiliaries using the AUXILIARY command in M*plus*. Variables that were significantly associated with study outcomes were included as controls.

## Results

### Sample characteristics

At the onset of the study, participants ranged from 19 to 44 years old (*M* = 32.09, SD = 4.27 years). Gender identity was assessed and all (100%) of respondents identified as “female.” A large portion of the sample identified as White (84.9%). The majority (97.7%) of participants reported completing education beyond high school. Participants reported a median annual household income range of $110,000–$149,999. Participants were between 4- and 26-weeks gestation (*M* = 21.44, SD = 8.93 weeks) and 52.6% were primiparous at T1. For comparative purposes, the current sample is slightly higher than the general Canadian population on income and education and is more homogeneous in terms of race (Statistics Canada 2017, 2022), but is similar to that of a Canada-wide COVID pregnancy sample (Lebel et al., 2020). On average, T2 surveys were completed 48.60 days (SD = 16.44) postpartum, T3 surveys were completed 6.56-months (SD = 0.65) postpartum, and T4 surveys were completed 15.52 months (SD = 0.69) postpartum. See Table 1 for additional sample characteristics.

**Table 1:** Sample characteristics and descriptive statistics.

	*n* (%)/*M* (SD)
Gestation	
First trimester	74 (24.3%)
Second trimester	137 (45.1%)
Third trimester	93 (30.6%)
Number of children	
0	144 (47.4%)
1	108 (35.5%)
2	40 (13.2%)
≥3	12 (4.0%)
Marital status	
Married	246 (80.9%)
Common-law	41 (13.5%)
In a relationship, but not married or	10 (3.3%)
Common law	2 (0.7%)
Divorced	1 (0.3%)
Separated single	4 (1.3%)
Race	
White	258 (84.9%)
Asian	21 (6.9%)
Indigenous	2 (0.7%)
Mixed race	9 (3.0%)
Other race	14 (4.6%)
Education	
Less than high school	1 (0.3%)
High school	11 (3.6%)
Non-university postsecondary	66 (21.7%)
University below bachelor’s	9 (3.0%)
Bachelor’s degree	117 (38.5%)
Above bachelor’s degree	99 (32.6%)
Annual family income	
<$20,000	3 (1.0%)
$20,000–$34,999	17 (5.6%)
$35,000–$69,999	33 (10.9%)
$70,000–$89,999	40 (13.2%)
$90,000–$109,999	47 (15.5%)
$110,000–$149,999	88 (28.9%)
$150,000–$199,999	46 (15.1%)
≥200,000	23 (7.6%)
Negative impact of COVID-19T1	3.36 (0.70)
Social Support (MSPSS) T1	5.60 (1.20)
Depression (CES-D) T1	11.49 (6.35)
Depression (CES-D) T2	11.62 (6.60)
Depression (CES-D) T3	10.64 (6.15)
Depression (CES-D) T4	10.55 (6.32)
Anxiety (GAD) T1	7.27 (5.07)
Anxiety (GAD) T2	8.26 (5.41)
Anxiety (GAD) T3	7.31 (4.95)
Anxiety (GAD) T4	6.70 (4.83)
Perceived Stress (PSS) T1	19.71 (7.02)
Perceived Stress (PSS) T2	20.94 (7.17)
Perceived Stress (PSS) T3	19.21 (7.34)
Perceived Stress (PSS) T4	18.16 (7.03)

Gestation at T1; CES-D = Center for Epidemiologic Studies Depression Scale; GAD = Generalized Anxiety Disorder-7 Questionnaire; T1 = pregnancy; T2 = 6-weeks postpartum; T3 = 6-months postpartum; T4 = 15-months postpartum; Negative impact of COVID-19 T1 = combined scale averaging subjective and objective COVID-19 impact; MSPSS = Multidimensional Scale of Perceived Social Support.

### Descriptive statistics

**Figure 1:**
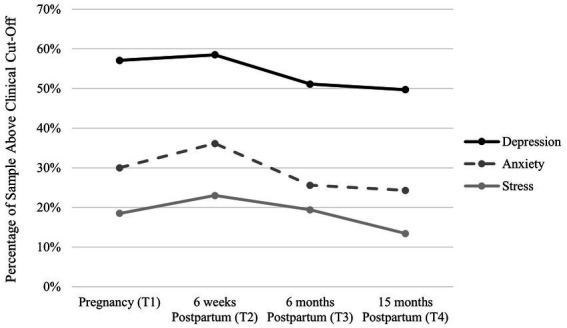
Percentage of the sample above the clinical cut-off for depression and anxiety across time.

See Table 1 for descriptive results for the distress, social support, and negative impact of COVID-19 scales. Rates of self-reported depression, anxiety, and perceived stress symptoms above established scale cut-offs are depicted in Figure 1. As shown in Figure 1, at T1, 57.1% of the sample scored ≥10 on the CES-D, indicating clinically significant levels of depression. The percentage of the sample scoring above the CES-D cut off was 58.5% at T2, 51.1% at T3, and 49.7% at T4. Similarly, moderate to severe anxiety (≥10 on the GAD-7) was endorsed by 30.0% of the sample at T1, 36.1% at T2, 25.6% at T3, and 24.3% at T4. Furthermore, high levels of stress (≥27 on the PSS) was endorsed by 18.5% of the sample at T1, 23.0% at T2, 19.4% at T3, and 13.4% at T4. These cut-off scores, in combination with the mean scores (Table 1), indicate that depression, anxiety, and perceived stress are slightly higher immediately after birth, and then decline slightly at 6-months and 15-months postpartum. Importantly, these descriptive results indicate that the average level of distress (depression, anxiety, and stress) reported at all time points in this sample are markedly higher than rates of distress reported in pregnant and postpartum samples prior to the COVID-19 pandemic (e.g., Ali, 2018; Liu et al., 2021).

### Preliminary bivariate correlations

As shown in Table 2, income and education level were correlated with depression, stress, and anxiety scores across time. Given the moderate correlation between education and income (*r* = 0.46, *p* < 0.01), only education was retained as a covariate in subsequent LGCMs, as it was more strongly correlated with distress measures. Although gestational age in pregnancy (T1) and relationship status were associated with longitudinal attrition, neither variable was significantly correlated with the study outcomes and were therefore treated as auxiliary variables.

**Table 2:** Preliminary correlations: Outcome variables and potential covariates.

	1	2	3	4	5	6	7	8	9	10	11	12	13	14	15	16	17	18	19	20	21
1. CESD T1																					
2. CESD T2	0.624^**^																				
3. CESD T3	0.575^**^	0.561^**^																			
4. CESD T4	0.562^**^	0.520^**^	0.628^**^																		
5. GAD T1	0.759^**^	0.514^**^	0.535^**^	0.497^**^																	
6. GAD T2	0.512^**^	0.729^**^	0.569^**^	0.479^**^	0.558^**^																
7. GAD T3	0.442^**^	0.390^**^	0.690^**^	0.401^**^	0.518^**^	0.597^**^															
8. GAD T4	0.553^**^	0.495^**^	0.632^**^	0.708^**^	0.574^**^	0.615^**^	0.556^**^														
9. PSS T1	0.839^**^	0.512^**^	0.555^**^	0.521^**^	0.722^**^	0.428^**^	0.476^**^	0.506^**^													
10. PSS T2	0.588^**^	0.783^**^	0.554^**^	0.563^**^	0.549^**^	0.663^**^	0.492^**^	0.500^**^	0.587^**^												
11. PSS T3	0.547^**^	0.546^**^	0.769^**^	0.572^**^	0.515^**^	0.635^**^	0.740^**^	0.603^**^	0.584^**^	0.668^**^											
12. PSS T4	0.545^**^	0.501^**^	0.580^**^	0.784^**^	0.480^**^	0.496^**^	0.518^**^	0.687^**^	0.582^**^	0.600^**^	0.675^**^										
13. Age	0.044	−0.020	−0.075	0.028	−0.031	−0.100	−0.124	−0.051	0.030	−0.054	−0.081	0.037									
14. Race	0.089	0.041	0.052	−0.034	0.047	0.047	0.010	0.026	0.098	0.047	0.114	0.082	−0.009								
15. Relation-ship	−0.096	0.030	−0.004	−0.057	−0.087	−0.019	−0.026	−0.049	−0.085	−0.063	−0.080	−0.094	−0.033	0.063							
16. Income	−0.189^**^	−0.106	−0.179^*^	−0.162^*^	−0.226^**^	−0.087	−0.109	−0.137	−0.210^**^	−0.165^**^	−0.160^*^	−0.237^**^	0.288^**^	−0.044	0.144*						
17. Education	−0.323^**^	−0.278^**^	−0.301^**^	−0.341^**^	−0.249^**^	−0.207^**^	−0.236^**^	−0.316^**^	−0.290^**^	−0.243^**^	−0.272^**^	−0.332^**^	0.169^**^	0.150^**^	0.220**	0.460^**^					
18. Offspring	0.049	0.050	0.031	0.177^*^	−0.001	−0.025	−0.045	0.051	0.054	0.016	−0.004	0.096	0.305^**^	−0.108	−0.236**	0.016	−0.097				
19. Gestation	−0.019	0.001	0.035	0.036	−0.077	0.052	−0.112	−0.058	−0.021	−0.021	−0.067	−0.020	0.021	0.053	−0.020	−0.006	0.000	0.060			
20. State of emergency	0.000	−0.005	−0.069	−0.082	0.036	0.034	−0.064	0.033	−0.033	−0.032	−0.013	−0.037	0.105	0.071	−0.029	0.097	0.122*	0.026	−0.096		
21. Gestational diabetes	−0.005	−0.018	−0.009	−0.016	−0.052	−0.081	−0.003	−0.084	−0.004	−0.011	−0.055	−0.042	0.075	0.013	−0.034	−0.070	−0.061	−0.052	−0.003	−0.069	
22. Miscarriage	0.023	−0.053	–	–	−0.049	−0.095	–	–	−0.030	−0.068	–	–	−0.015	−0.024	0.009	0.060	0.013	0.015	0.042	0.067	−0.017

CESD = Center for Epidemiologic Studies Depression Scale (CES-D); T1 = pregnancy; T2 = 6-weeks postpartum; T3 = 6-months postpartum; T4 = 15-months postpartum; GAD = Generalized Anxiety Disorder-7 Questionnaire (GAD-7); race = dichotomous (White = 0, non-White = 1); Relationship = Relationship status at T1 (dichotomized 1 = in a relationship, 0 = not in a relationship); offspring = number of children, not including current pregnancy; Gestation = number of weeks gestation at the onset of the study (T1); State of emergency = number of days since the initial State of Emergency in Ontario at the onset of the study (T1); Gestational diabetes = dichotomous (yes = 1, no = 0); Miscarriage = dichotomous (yes = 1, no = 0). – = correlation cannot be computed because one variable is constant (this is a result of only *n* = 1 reporting experiencing a miscarriage).

### Longitudinal measurement model

The initial longitudinal CFA model provided an acceptable fit to the data, *χ*^2^ (30) = 80.74, *p* < 0.001, RMSEA = 0.075, CFI = 0.983, SRMR = 0.041. The three indicators loaded strongly onto the latent factors at each time point (depression *β*s = 0.88–0.94; anxiety *β*s = 0.77–0.81; stress *β*s = 0.86–0.89). Individual differences in latent distress showed a moderate to strong degree of stability over time (*r*s = 0.66–0.72). Constraining the item loadings (ΔCFI = 0.002, ΔRMSEA = −0.01) and intercepts (ΔCFI = 0.005, ΔRMSEA = 0.003) to equality over time did not lead to substantive decreases in model fit, thus providing support for the assumption of measurement invariance.

### Unconditional LGCMs

The initial linear model provided a relatively poor fit to the data *χ*^2^ (48) = 155.03, *p* < 0.001, RMSEA = 0.086, CFI = 0.968, SRMR = 0.093, whereas the piecewise LGCM provided an acceptable fit the data, *χ*^2^ (44) = 112.24, *p* < 0.001, RMSEA = 0.072, CFI = 0.979, SRMR = 0.052, and represented a significant improvement over the linear model, Δ*χ*2 (4) = 41.02, *p* < 0.001, ΔRMSEA = −0.014, ΔCFI = 0.011. Parameter estimates for the piecewise LGCM are displayed in Table 3. As shown in Table 3, average levels of distress remained relatively similar from the T1 to T2 and then declined significantly from T2 to T4. As shown in Table 4, the distress intercept was significantly correlated with slope 1, but not slope 2, and slopes 1 and 2 were not correlated. Thus, distress levels during pregnancy were associated with the rate of change in distress between pregnancy and 6-weeks postpartum, but not the rate of change in distress from 6-weeks postpartum to 15-months postpartum. In addition, correlations between intercepts and slopes and predictors/covariates in the unconditional model are displayed in Table 4. As shown in Table 4, the intercept was significantly negatively correlated with education and social support, indicating that those with higher social support and higher income reported lower levels of distress at T1. The intercept was also positively correlated with negative COVID impact, indicating that those who reported the pandemic to have a more negative impact also reported higher levels of distress at T1. Slope 1 was significantly positively correlated with social support and negatively correlated with negative COVID impact. Education, social support, and negative covid impact were not significantly correlated with slope 2. Thus, the regression paths between these variables and slope 2 were constrained to zero in the final conditional piecewise LGCM.

**Table 3:** Unconditional parameter estimates for the piecewise growth model.

	Unst. Coeff.	SE	Value of *p*
Int mean	2.455	0.067	<0.001
Int variance	1.199	0.089	<0.001
S1 mean	0.108	0.058	0.065
S1 variance	0.454	0.134	0.001
S2 mean	−0.162	0.033	<0.001
S2 variance	0.060	0.053	0.257

In order to constrain depression and anxiety paths to be equivalent, both depression and anxiety scores were rescaled to range from 1 to 7. Dep = depression; Anx = anxiety; Int = intercept; S1 = slope 1; S2 = slope 2.

**Table 4:** Correlations between intercepts, slopes and predictors.

	1	2	3	4	5
1. Intercept					
2. Slope 1	−0.373***				
3. Slope 2	−0.026	−0.055			
4. Education	−0.501***	0.070	−0.044		
5. Social support	−0.507***	0.156*	−0.059	0.353**	
6. COVID Impact	0.470***	−0.164**	−0.014	−0.213**	−0.133*

S1 = slope 1; S2 = slope 2. *p < 0.05, **p < 0.01, ***p < 0.001.

### Conditional piecewise LGCM

In the final model, shown in Table 5, higher education, greater social support, and lower negative impact of COVID-19 were uniquely associated with lower average distress intercept (during pregnancy). Unexpectedly, having greater social support was also associated with a greater increase in distress from pregnancy to 6-weeks postpartum (slope 1). In addition, more negative impact of COVID-19 was associated with a faster decrease in distress from pregnancy to 6-weeks postpartum (slope 1). It is important to note that those who reported higher levels of social support experienced lower levels of distress during pregnancy. In fact, when we controlled for the effect of the distress intercept on slope 1 (*b* = −0.369, *p* < 0.001), social support and COVID-19 impact were no longer significant predictors of slope 1 (*p*s = 0.94–97). Thus, even though social support and COVID impact predicted a greater increase in distress symptoms over time, this effect is likely due to those individuals having lower levels of distress at T1. We also explored whether social support moderated the association between initial levels of distress and rate of change in distress, but this result was not significant.^1^

**Table 5:** Regression estimates from final piecewise growth model.

	Unst. Coeff.	Std Coeff.	Std SE	Std *p-*value	Std 95% CI
Int					
Education	−0.151	−0.190	0.046	<0.001	−0.281, −0.100
Social support	−0.264	−0.289	0.058	<0.001	−0.404, −0.175
COVID impact	0.573	0.440	0.048	<0.001	0.346, 0.534
S1					
Education	−0.002	−0.005	0.093	0.957	−0.189, 0.179
Social support	0.094	0.181	0.089	0.041	0.007, 0.355
COVID impact	−0.209	−0.283	0.086	0.001	−0.450, −0.115

Int = intercept of distress latent variable; S1 = slope 1 (pregnancy to T2) of distress latent variable; Unst. = unstandardized; Std = standardized; Coeff = coefficient.

## Discussion

This is the first study, to our knowledge, to document levels of maternal distress during pregnancy and the postpartum period, over several time points across 2 years of the COVID-19 pandemic. Descriptive results demonstrate markedly high levels of self-reported depressive symptoms, anxiety, and perceived stress, across the perinatal period up to 15-months postpartum. Results of the latent growth curve models indicate that average levels of distress remained relatively stable (although descriptively, we observe a slight increase) from pregnancy to 6-weeks postpartum and then declined significantly from 6-weeks postpartum to 15-months postpartum. We found that lower negative impact of COVID-19 and greater social support were associated with lower levels of distress in pregnancy. These factors were also associated with the rate of change in distress symptoms from pregnancy to 6-weeks postpartum, such that participants with greater social support and less negative COVID impact, had greater increase in distress symptoms up to 6-weeks postpartum. This counterintuitive finding will be discussed in more detail below. Taken together, the findings of this longitudinal study highlight the changing nature of distress symptoms experienced by pregnant and postpartum people over 2 years of the COVID-19 pandemic, as well as factors that impact the severity of this distress.

### Distress levels across time: Pregnancy to 15-months postpartum

On average, across the four timepoints (during pregnancy and postpartum) of this study, 50–58% of the sample self-reported clinically significant levels of depression, 24–36% of the sample reported moderate to severe anxiety, and high levels of stress were reported by 13–18% of the sample. These findings indicate substantially higher average scores of distress in comparison to pre-COVID pregnant and postpartum samples. A meta-analysis estimated the global prevalence of postpartum depression to be 14% (Liu et al., 2021), which is consistent with estimates from the American Psychological Association (2008). The higher rates of depression observed here are in line with a recent meta-analysis of postpartum depression during COVID-19, which showed that studies using the Edinburgh Postnatal Depression Scale (EPDS) cut off scores between 10 and 12, reported between 27 and 44% of the sample scoring above the cut off (Safi-Keykaleh et al., 2022). In addition, typical estimates of generalized anxiety range between 3 and 10% during pregnancy (Misri et al., 2015; Viswasam et al., 2019) and 4–11% in the postpartum period (Misri et al., 2015; Ali, 2018). A recent meta-analysis estimated antenatal anxiety symptoms to be experienced by ~40% of samples and up to 56% in European samples during COVID-19 (Shorey et al., 2021). These findings, in line with prior research, highlight the continued need to support pregnant and postpartum people, as these individuals are continuing to experience elevated rates of distress as the COVID-19 pandemic wages on.

In addition, the present findings demonstrate a slight (but not significant) increase in distress from pregnancy to 6-weeks postpartum. Similarly, prior longitudinal research conducted during the pandemic reported elevated levels of depression from pregnancy to the first few months of postpartum (Duguay et al., 2022; Gluska et al., 2022). Research prior to the pandemic identified 12 weeks postpartum as the period with the highest prevalence of postpartum depression, with a marked decline thereafter (Liu et al., 2021). We also observed a decline in distress ratings from 6-weeks postpartum to 15-months postpartum, which is consistent with pre-COVID research and is indicative of normative changes in distress across the postpartum period (Putnick et al., 2020). However, the decline in distress later in the postpartum period observed in this study are not such that these levels compare to pre-pandemic population levels.

We also observed nonsignificant associations between the rates of change in distress levels over the perinatal period and the amount of variability in distress later in the postpartum. First, the rate of change in distress from pregnancy to 6-weeks postpartum was not associated with the rate of change from 6-weeks to 15-months postpartum. One potential explanation for this is childbirth and the transition to parenthood. It is possible that this transition disrupted/changed any ongoing trajectories of distress. Second, we did not observe significant variability in the rate of change in distress from 6-weeks to 15-months postpartum (the way in which distress changed tended to be similar across the sample). This may be due to the relatively modest sample size or sample collection points. Limited variability in the rate of change in distress symptoms, from 6-weeks postpartum to 15-months postpartum, likely explains the nonsignificant predictors of the change in distress later in the postpartum period.

### Factors related to distress: Education level, COVID impact and social support

Findings indicate that greater negative COVID impact, lower education, and lower social support were positively associated with higher levels of distress levels during pregnancy. These findings are in line with prior research indicating that social support is a protective factor (e.g., Lebel et al., 2020; Zhou et al., 2021; Khoury et al., 2021b; Fernandes et al., 2022) and COVID-related stress is a risk factor (e.g., Khoury et al., 2021b; Awad-Sirhan et al., 2022; Giesbrecht et al., 2022) for mental health and distress in prenatal and postpartum samples during the pandemic. In particular, prior work by Fernandes et al. (2022) showed that higher social support was associated with lower levels of depression from pregnancy 6-months postpartum. The present study adds to this literature by demonstrating longitudinal effects of social support and COVID stress on trajectories of multiple indices of distress (depression, anxiety, and stress) from pregnancy to 15-months postpartum. Specifically, in addition to impacting prenatal levels of distress, we found that higher social support and lower negative COVID impact were associated with a greater increase in distress from pregnancy to 6-weeks postpartum. It is important to consider the role of pregnancy (baseline) levels of distress, such that participants reporting higher levels of social support (and less negative COVID impact), also reported lower levels of distress during pregnancy, and the effects of social support and COVID impact on trajectories of distress were no longer significant once pregnancy levels of distress were accounted for. This underscores the importance of prenatal distress levels on setting the trajectories of distress throughout the postpartum period, and the need to bolster protective factors and reduce risk factors, during pregnancy, as it has importance for distress levels throughout the postpartum period. Perinatal mental health and distress impact both maternal wellbeing and offspring developmental outcomes (for reviews see Kingston et al., 2012; Kingston and Tough, 2014; Caparros-Gonzalez et al., 2021), as is beginning to become evident during the COVID-19 pandemic (e.g., Preis et al., 2021; Khoury et al., 2022). Thus, it is essential to mitigate risk associated with maternal distress during the perinatal period.

The present findings also underscore the impact SES-related factors (education, income) on distress during the pandemic. In our study, despite having a relatively low-risk sample in terms of family income and maternal education level, those with lower education (and lower income) were observed to have higher levels of distress in pregnancy. This is in line with prior research demonstrating the unequal negative impact of the pandemic on low SES individuals and families (Baena-Díez et al., 2020; Reimer et al., 2021; Spinelli et al., 2021) and racial minorities (Park, 2021; Perrigo et al., 2022). In fact, SES is shown to impact parenting attitudes and activities associated with cognitive development in infants and toddlers (Hendry et al., 2022) as well as older children (Stienwandt et al., 2022) during the pandemic.

### Practical implications: Need for mental health and social support

We demonstrate that heightened distress during the COVID-19 pandemic is linked to persistent, clinically significant levels of depression, anxiety and perceive stress during pregnancy and beyond the first year postpartum. These findings strengthen the appeal for enhanced mental health support and easily accessible social support services for pregnant and postpartum individuals, especially during times of increased isolation, such as the COVID-19 pandemic. Given these findings, programs aimed to bolster social support for all pregnant individuals, have the potential to buffer against adverse parent mental health outcomes, which can affect the parent and child (Ohara et al., 2017; Feinberg et al., 2022). The intergenerational risk to child wellbeing can be protected through parent mental health support and intervention (Thanhäuser et al., 2017). There is an urgent need to develop easily accessible programming in anticipation of future – possibly long-lasting – public health crises. The move toward affordable and easily accessible telehealth mental health services can reduce a number of barriers to seeking services often experienced by perinatal women and parents. Easily accessible services can benefit those who are at sociodemographic risk and, in turn, greater risk for experiencing elevated distress. Considerations that make mental health and social support more easily accessible for families in need is essential for parents and families to begin to recover from the pandemic.

### Strengths and limitations

This study adds to the existent literature regarding perinatal psychological distress, as well as COVID-19 specific distress during pregnancy and the postpartum period. A strength of this research is the extended longitudinal design, including perinatal mental health assessments at four time points across 2 years of the pandemic. However, the current study is not without limitations. Firstly, the sample is considered socio-demographically low-risk, as many participants were well educated and reported high income. Research with more diverse samples is needed to determine if similar distress trajectories are found in samples who report lower socioeconomic factors. Additionally, this study exclusively used self-report measures of distress, which, due to the subjective nature of individual differences in the appraisal of stressful events, may impact the comparability of distress ratings. Exclusive reliance on self-report instruments can also potentially contribute to overestimation of symptomatology. Future research would benefit from a multi-method approach (clinical interviews and self-report). In addition, although prior research indicates that variations in distress across trimesters of pregnancy during the pandemic (Bérard et al., 2022), the current study is limited by varied pregnancy trimester at T1 (though weeks gestation was not a significant covariate). In addition, while the study sample size remained relatively consistent at T3 and T4 (*n* = 180, *n* = 190, respectively), there was attrition from T1 to T3 (T1 *n* = 304, T2 *n* = 265), with 63% of the original sample retained at T4. This attrition reflects the challenge of maintaining a longitudinal cohort throughout the pandemic, amidst the numerous stressors these families faced. As is standard practice (Collins et al., 2001), missing data was accounted for statistically and all results are based on the full sample. Lastly, it should be noted that due to the nature of this research starting early in the pandemic, pre-pandemic measures of mental health symptoms were not obtained, therefore limiting the ability to assess pre- to post-pandemic changes in distress.

## Conclusion

This study extends prior research by demonstrating that perinatal individuals are continuing to experience high levels of depression, anxiety, and stress from pregnancy up to 15-months postpartum, during the COVID-19 pandemic. Findings also highlight the importance of social support and negative impact of COVID-19 as potentially modifiable targets to reduce perinatal levels of distress. These findings underscore the essential need to improve mental health support for perinatal individuals and their families, because although daily life has resumed to normalcy in many ways, the mental health impact of COVID-19 continues.

## Data availability statement

The data analyzed in this study is subject to the following licenses/restrictions: Data can be made available upon reasonable request to the corresponding author. Requests to access these datasets should be directed to jennifer.khoury@msvu.ca.

## Ethics statement

The studies involving human participants were reviewed and approved by the Hamilton Integrated Research Ethics Board and the Mount Saint Vincent University Research Ethics Board. Written informed consent to participate in this study was provided by the participants’ legal guardian/next of kin.

## Author contributions

JK, AG, and LA designed the study and wrote the protocol. JK and LG carried out the data collection and management. JK and MJ undertook the statistical analysis and writing the results. JK wrote the first draft of the manuscript. All authors contributed to the article and approved the submitted version.

## Funding

This work was funded by a Canadian Institute of Health Research (CIHR) Project Grant – PA: Pandemic and Health Emergencies Research (465280). This work was also supported by a Tier II Canadian Research Chair (CRC) in Interdisciplinary Studies in Neurosciences awarded to JK and a Tier II CRC in Family Health and Preventive Interventions awarded to AG.

## Conflict of interest

The authors declare that the research was conducted in the absence of any commercial or financial relationships that could be construed as a potential conflict of interest.

## Publisher’s note

All claims expressed in this article are solely those of the authors and do not necessarily represent those of their affiliated organizations, or those of the publisher, the editors and the reviewers. Any product that may be evaluated in this article, or claim that may be made by its manufacturer, is not guaranteed or endorsed by the publisher.

## Footnotes

^1^Social support did not significantly moderate the relation between initial levels of distress and rate of change in distress (Slope 1: beta = −0.008, SE = 0.038, *p* = 0.831; Slope 2: beta = −0.020, SE = 0.026, *p* = 0.438). Thus, although social support was independently related to initial levels of distress and change in distress (slope 1), it did not influence the association between distress at T1 and change in distress. This non-significant moderation may be due to the strong association between distress across time or limited power in our analyses.
