# Supplementary material for: Trajectories of distress from pregnancy to 15-months post-partum during the COVID-19 pandemic
Source: Front Psychol. 2023 Mar 31;14:1104386. doi: 10.3389/fpsyg.2023.1104386 (PMC10102331; doi:10.3389/fpsyg.2023.1104386)
Supplement: Supplementary file 1 [file Table_1.docx]

Supplementary Table 1

*Demographic* *characteristics of participants who completed and did not complete the study*

|  | **Completed T1-T4**  **N(%)/M(SD)** | **Did not complete T2**  **N(%)/M(SD)** | **Did not complete T3**  **N(%)/M(SD)** | **Did not complete T4**  **N(%)/M(SD)** |
| --- | --- | --- | --- | --- |
| **Sample Size (n)** | N = 151 | N = 39 | N = 124 | N = 122 |
| **Gestational age at T1** | 22.25 (8.48) | 18.08 (9.01) | 20.10 (9.22) | 20.15 (9.65) |
| **Maternal age** | 32.33 (3.96) | 32.59 (5.26) | 31.67 (4.42) | 31.79 (4.83) |
| **Number of children**  0  1  2  > 3 | 66 (43.71)  62 (41.06)  19 (12.58)  4 (2.65) | 22 (56.41)  8 (20.51)  5 (12.82)  4 (10.26) | 65 (52.42)  34 (27.42)  18 (14.52)  7 (5.65) | 64 (52.46)  37 (30.33)  14 (11.48)  7 (5.74) |
| **Relationship status**  In a romantic relationship  Not in a romantic relationship | 150 (99.34)  1 (0.66) | 37 (94.9)  2 (5.1) | 118 (95.2)  6 (4.8) | 117 (95.90)  5 (4.10) |
| **Education**  Less than high school  High school  Trade certificate or diploma  Non-university postsecondary  University below Bachelor’s degree  Bachelor’s degree  Above Bachelor’s degree | 0 (0.00)  2 (1.33)  2 (1.33)  29 (19.33)  4 (2.67)  60 (40.00)  53 (35.33) | 0 (0.00)  2 (5.13)  0 (0.00)  6 (15.38)  0 (0.00)  18 (46.15)  13 (33.33) | 1 (0.81)  8 (6.45)  3 (2.42)  24 (19.35)  5 (4.03)  47 (37.90)  36 (29.03) | 1 (0.82)  7 (5.74)  3 (2.46)  23 (18.85)  4 (3.28)  47 (38.52)  37 (30.33) |
| **Annual Family Income**  < $20,000  $20,000 to $34,999  $35,000 to $69,999  $70,000 to $89,999  $90,000 to $109,999  $110,000 to $149,999  $150,000 to $199,999  > 200,000 | 0 (0.00)  5 (3.43)  13 (8.90)  17 (11.64)  24 (16.44)  46 (31.51)  26 (17.81)  15 (10.27) | 0 (0.00)  4 (10.26)  4 (10.26)  4 (10.26)  6 (15.38)  12 (30.77)  7 (17.95)  2 (5.13) | 3 (2.42)  10 (8.06)  15 (12.10)  20 (16.13)  22 (17.74)  27 (21.77)  18 (14.52)  7 (5.65) | 3 (2.50)  9 (7.50)  16 (13.22)  21 (17.50)  16 (13.33)  35 (29.17)  13 (10.83)  7 (5.83) |
| **Race**  White  Asian  Indigenous  Mixed Race  Other Race | 131 (86.76)  10 (6.62)  1 (0.66)  5 (3.31)  4 (2.65) | 30 (76.92)  3 (7.69)  0 (0.00)  3 (7.69)  3 (7.69) | 102 (82.26)  10 (8.06)  1 (0.81)  3 (2.42)  8 (6.45) | 97 (79.51)  10 (8.20)  1 (0.82)  4 (3.28)  10 (8.20) |
| **Depressive symptoms (T1-T4 average)** | 10.98 (5.19) | 11.43 (6.08) | 11.64 (5.95) | 11.63 (5.98) |
| **Anxiety symptoms (T1-T4 average)** | 7.49 (4.11) | 6.53 (5.23) | 7.33 (4.76) | 7.35 (4.71) |
| **Perceived Stress (T1-T4 average)** | 19.56 (6.08) | 19.81 (7.20) | 20.19 (6.45) | 20.31 (6.46) |

*Note:* T1 = pregnancy, T2 = 6 weeks postpartum, T3 = 6 months postpartum, T4 = 15 months postpartum. Participants who completed T2 did not differ from those who did not on parent age, race, ethnicity, income, or number of children. However, participants who entered the study earlier in their pregnancy were less likely to complete T2 (*t*(302) = 2.54, *p* < .05), T3 (*t*(302) = 2.17, *p* < .05), and T4 (*t*(302) = 2.07, *p* < .05). Participants who had lower income were also less likely to complete T3 (*t*(295) = -2.91, *p* < .01) and T4 (*t*(295) = -3.03, *p* < .01). Lastly, those who were not in a romantic relationship (*t*(302) = 2.46, *p* < .05) were also less likely to complete T3. No other significant differences were found in participants who completed all time points and those who did not. Participants who completed all assessments did not differ from those who did not complete T2-T4 on perceived stress, anxiety symptoms, depressive symptoms, social support or COVID stress at T1 (*p*s range .07-.86).
